# Lung Epithelial Protein Expression and the Use of Volatile Anesthetics in Acute Respiratory Distress Syndrome

**DOI:** 10.7759/cureus.10196

**Published:** 2020-09-02

**Authors:** Eric P Decker, Audrey A Vasauskas

**Affiliations:** 1 Anesthesiology, Alabama College of Osteopathic Medicine, Dothan, USA; 2 Molecular Medicine, Alabama College of Osteopathic Medicine, Dothan, USA

**Keywords:** volatile anesthetics, acute respiratory distress syndrome, lung epithelium

## Abstract

Acute respiratory distress syndrome (ARDS) is a potentially fatal lung injury that can present with divergent underlying cause across cases. Current treatment options are limited by an incomplete understanding of the disease sequelae, undefined unifying pathology, and lack of reliable diagnostic tools. ARDS is defined as respiratory failure not caused by fluid overload or cardiac failure within one week of a known clinical insult with bilateral opacities on chest imaging, and diagnosis is based on these parameters. Increased understanding of the inflammatory cascade associated with ARDS progression shows promise for identifying potential diagnostic biomarkers and additional treatment options. Here, we review recent studies that point to the unifying inflammatory element(s) of the disease process and the use of agents that decrease inflammation as potentially powerful treatments for ARDS patients.

## Introduction and background

Introduction

Acute respiratory distress syndrome (ARDS) is an incompletely understood process describing life-threatening lung injury with a variety of underlying causes. It is defined as respiratory failure not caused by fluid overload or cardiac failure, within one week of a known clinical insult, with bilateral opacities visible on chest imaging [1]. Treatment of ARDS is currently limited with no effective pharmacological intervention and limited management options focused primarily on lung protective mechanical ventilation and treatment of the underlying cause. Similarly, we lack a reliable biomarker to test for ARDS meaning the diagnosis is based primarily on clinical suspicion along with imaging [2].

Here, we review the recent studies which have sought to address this gap in knowledge with the majority showing ARDS results in increases to primarily inflammatory markers with upregulation of genes also normally seen in an inflammatory response. Increases have been seen with IL-6, IL-1, histones, and receptor for advanced glycation end-products (RAGE) levels all of which have clinical significance and present the possibility of expanded treatment options [2]. Specifically, the inflammatory response seen in ARDS suggests the need for the maintenance of sedation for intubated patients with agents that decrease inflammation such as inhaled volatile anesthetics (VA) [3]. Identification of further biomarkers of ARDS and more specific therapies for its treatment may result in a decreased reliance on ventilators, shortened intubation time, and decreased mortality.

Methodology

A systematic review of literature was performed using Pubmed. Keywords included “ARDS biomarkers”, “ARDS protein expression”, and “ARDS RNA expression”. Studies were included from the last five years with a focus on novel pathways implicated in the ARDS pathogenesis. Studies were excluded if they were from journals not written in English as well as those which presented information already readily available in the knowledge base.

ARDS background

ARDS is relatively common in patients admitted to an intensive care unit (ICU) with a prevalence of around 10%. Additionally, its mortality rate is extremely high at roughly 30% during hospitalization and 41% over the course of a year following initial diagnosis, although this has been improving with recent changes in treatment over the last decade [4]. Such treatment typically involves prone positioning, high positive end-expiratory pressure, low volume, high-frequency respiration, conservative fluids, and occasionally steroids [5]. Additionally, any medical treatment beyond this focuses almost exclusively on the underlying cause of ARDS. ARDS is defined by the Berlin definition as lung injury within one week of a known insult, with bilateral opacities present on chest CT and x-ray, not explained by heart failure or fluid overload [1]. We currently lack a well-tested biomarker that can be readily used in clinical practice to diagnosis or monitor the resolution of ARDS [2]. Past research has shown increases in inflammatory markers such as tumor necrosis factor (TNF) and interleukins 1, 6, and 10 although none of these markers are specific to ARDS and do not serve as a reliable diagnostic tool [6].

The pathophysiology of ARDS is characterized by damage to the capillary endothelium and alveolar epithelium by toxic mediators released by neutrophils [5]. This neutrophil-mediated damage to capillary endothelium results in a breakdown of the normal lymphatics of the lung with subsequent overwhelming edema. Consequences of ARDS include pulmonary hypertension (although it rarely results in right-sided heart failure for unknown reasons), pulmonary fibrosis, and long-term increases in mortality [7]. Sedation of patients with ARDS is typically achieved via propofol in the United States; however, alternatives which suppress neutrophil function, such as sevoflurane, have been shown to decrease the length of hospitalization in patients with ARDS [3].

## Review

Protein expression and biomarkers of ARDS

Recent studies have shown ARDS pathophysiology to be more diverse than originally thought involving multiple additional pathways. Both neutrophils and type I lung epithelial cells possess RAGE [6]. When bound to its ligand, RAGE triggers a complex downstream pathway which ultimately results in the activation of nicotinamide adenine dinucleotide phosphate (NADPH) oxidase which is known to be responsible for the damage to the capillary/alveoli barrier characteristic of ARDS [8]. While attached to cells RAGE is pro-inflammatory and plays an important role in the ARDS disease process. However, when free, in blood, it binds excess advanced glycation products, thereby reducing inflammation 6].

Histones, specifically H3 and H4, may be generated in the extracellular space as a result of the initial insult leading to an increase in inflammation. This is mediated through binding of these extracellular histones to toll-like receptors (TLR) 2, 4, and 9 [9]. Additionally, histones may directly activate the NLRP3 inflammasome which has been linked to the neutrophil infiltration and albumin leakage characteristic to ARDS [9].

Blood work from patients with ARDS shows that levels of a variety of molecules correspond with disease severity including IL-8, surfactant protein D, angiopoetin-2, and RAGE. Additionally, bronchoalveolar lavage shows increased levels of Fas, Fas ligand, PCP I, and PCP III. Current studies have attempted to evaluate if any of these molecules are specific and sensitive enough to diagnose ARDS [10]. All of these molecules are candidates for use as biomarkers and may provide a more reliable diagnosis especially when multiple molecules are used simultaneously.

The current limited understanding of ARDS has resulted in treatment options that leave much to be desired. Both RAGE and extracellular histones can be specifically targeted with promising results seen in vitro for the treatment of ARDS. Both of these markers have been linked to the increase in inflammation seen in ARDS. Multiple molecules have been considered for use as a definitive biomarker to allow for monitoring for resolution of ARDS including RAGE, Il-8, and angiopoetin-2 although none have been reliably tested in vivo to determine which is best for screening [2].

Treatment of ARDS with volatile anesthetics

Traditionally patients with ARDS in the United States who require mechanical intubation have been treated in ICU with the primary sedative of propofol along with benzodiazepines, ketamine, and alpha-2 antagonists. This is in contrast with our European colleagues who instead may rely on volatile anesthetics (VAs) to maintain the required sedation necessary for patients to remain intubated [11]. This European model has been shown to have cost-benefit as well as being feasible for use in the United States [12]. There are multiple reasons that despite this inhaled anesthetics have not gained more use in American ICUs not least of which is that, the current respirators used in ICU in the United States lack the ability to use VAs due to using a non-circular system in which gases are not recycled after being exhaled by the patient and therefore enter the surrounding room. This is in contrast to anesthesia machines used in the operating room (OR) which are circular and as such do not result in the contamination of the outside air with the gases used to provide anesthesia [11]. Another important consequence of this difference is that recirculation of air for anesthesia machines will lead to self re-exposure of the patient to any virus which they are shedding through respiratory droplets [13].

VAs potentially have a significant benefit over the current IV sedatives used in the United States as they have been shown to result in less oxidative stress, lower proinflammatory markers, and an attenuation in neutrophil response over ketamine [11]. Particularly, sevoflurane resulted in the greatest reduction in inflammation. Studies in mice suggest that this occurs via inhibition of the PI3K/Akt signaling pathway [14]. This inhibition results in a decrease in both inflammation and lung permeability while maintaining the capillary-alveolar barrier damaged in ARDS. While potentially useful in ARDS with a known viral insult, this reduction in inflammation is undesired in cases where bacterial infection is present as it may impair the immune systems ability to respond appropriately, potentially prolonging infection [11]. As compared to a viral infection, a strong immune response is desired in cases of bacterial infection such that successful eradication of the bacterium may occur [11] Additionally, patients sedated with VAs have less time till extubation as compared to midazolam, another common IV anesthetic used in the United States [15]. This has significant benefits through decreasing hospital length of stay, lowering cost both to the hospital and patient, and reducing complications associated with prolonged mechanical ventilation [16].

## Conclusions

Summary and future work

With the recent COVID-19 pandemic, ARDS has become a daily concern for physicians around the country. While incompletely understood, recent studies have offered the possibility of both better treatment options and the ability to diagnose and monitor for resolution of ARDS using bloodwork. The clinical implications of this are massive as we are currently limited to treating the underlying cause of ARDS and offering supportive therapies to those with this potentially fatal syndrome. So far, the majority of studies have shown ARDS to be primarily an over inflammatory state with increases seen in a broad variety of inflammatory markers such as interleukin-8, extracellular histones, and RAGE. While there is some potential for each of these proteins to be used as a marker for ARDS, none currently have been established as reliable for a clinical setting either on their own or in a panel. Studies are beginning to utilize our progressing knowledge of the inflammatory response seen in ARDS to develop better treatment methodology as can be seen with the current trials using sevoflurane to maintain sedation in intubated ARDS patients.

Future research on this subject is broad and would largely consist of testing the various biomarkers and treatment options in a clinical setting. The use of sevoflurane is widespread outside of the United States and as such seems to be ready for clinical trials, especially in light of the recent severe acute respiratory syndrome coronavirus 2 (SARS-COV2) pandemic and the vast increase in virally mediated SARS with secondary cytokine storm. Additionally, studies that seek to identify which long-term physiological changes, if any, are present in the lung endothelium are of interest since the present literature does not have an explanation as to the rare occurrence of cor pulmonale following ARDS.
